# Dynamics of propagation of premature impulses in structurally remodeled infarcted myocardium: a computational analysis

**DOI:** 10.3389/fphys.2014.00483

**Published:** 2014-12-16

**Authors:** Candido Cabo

**Affiliations:** ^1^Department of Computer Systems, New York City College of Technology, City University of New YorkNew York, NY, USA; ^2^Doctoral Program in Computer Science, Graduate Center, City University of New YorkNew York, NY, USA

**Keywords:** premature impulses, cell-to-cell conductance, structural remodeling, conduction velocity restitution curve, infarction, computer model

## Abstract

Initiation of cardiac arrhythmias typically follows one or more premature impulses either occurring spontaneously or applied externally. In this study, we characterize the dynamics of propagation of single (S2) and double premature impulses (S3), and the mechanisms of block of premature impulses at structural heterogeneities caused by remodeling of gap junctional conductance (*Gj*) in infarcted myocardium. Using a sub-cellular computer model of infarcted tissue, we found that |I_Na,max_|, prematurity (coupling interval with the previous impulse), and conduction velocity (CV) of premature impulses change dynamically as they propagate away from the site of initiation. There are fundamental differences between the dynamics of propagation of S2 and S3 premature impulses: for S2 impulses |I_Na,max_| recovers fast, prematurity decreases and CV increases as propagation proceeds; for S3 impulses low values of |I_Na,max_| persist, prematurity could increase, and CV could decrease as impulses propagate away from the site of initiation. As a consequence it is more likely that S3 impulses block at sites of structural heterogeneities causing source/sink mismatch than S2 impulses block. Whether premature impulses block at *Gj* heterogeneities or not is also determined by the values of *Gj* (and the space constant λ) in the regions proximal and distal to the heterogeneity: when λ in the direction of propagation increases >40%, premature impulses could block. The maximum slope of CV restitution curves for S2 impulses is larger than for S3 impulses. In conclusion: (1) The dynamics of propagation of premature impulses make more likely that S3 impulses block at sites of structural heterogeneities than S2 impulses block; (2) Structural heterogeneities causing an increase in λ (or CV) of >40% could result in block of premature impulses; (3) A decrease in the maximum slope of CV restitution curves of propagating premature impulses is indicative of an increased potential for block at structural heterogeneities.

## Introduction

Many cardiac arrhythmias have a reentrant mechanism, a pattern of excitation in which a wave rotates around an anatomical or functional obstacle (Wit and Janse, 1993). It is well-established that reentrant arrhythmias require a “trigger,” which, in combination with a suitable “substrate,” creates the conditions for initiation of a reentrant wave (i.e., a wave break) (Wit and Janse, 1993). One of the conditions for initiation of reentrant arrhythmias is the creation of a region of unidirectional block, which, by allowing propagation of the impulse in some directions but not in others, leads to the creation of wave breaks (Kleber and Rudy, 2004). Unidirectional block can occur in homogeneous tissue (Frazier et al., 1989; Quan and Rudy, 1990), in tissues with spatial heterogeneities in cell properties (refractory period, membrane excitability) (Janse and Kleber, 1981; Gough et al., 1985; Coronel, 1994) or in tissues with discontinuities in the microstructure (cell size, gap junction conductance) (Toure and Cabo, 2012) or the macrostructure (muscle bundle branches, narrow isthmuses, pivot points, tissue expansions) (Joyner, 1981; Spach et al., 1982; Cabo et al., 1994, 1996; Fast and Kleber, 1995).

In experimental and clinical cardiac electrophysiology, the conditions for unidirectional block are created by external electrical stimulation using sequences of closely coupled (i.e., premature) impulses (the “trigger”). Premature impulses can also occur spontaneously in healthy and diseased hearts as a result of reentry, abnormal automaticity or triggered activity (Peters et al., 2000). In healthy hearts, premature impulses are often benign (i.e., do not result in unidirectional block and arrhythmias) but, the structural and/or membrane remodeling caused by heart disease may lead to the creation of a suitable “substrate” such that premature impulses may result in unidirectional block and the initiation of arrhythmias (Nattel et al., 2007).

Structural remodeling following heart disease can cause local heterogeneities in the tissue microstructure, like an increase in cell size (hypertrophy) (Nattel et al., 2007) and remodeling of connexin43 (Cx43) (Severs et al., 2008). Myocardial infarction results in a reduction of the amount of Cx43 with the consequent decrease in gap junction conductance and conduction velocity (Cabo et al., 2006). Regions of heterogeneous Cx43 expression and gap junction conductance have been described in infarcted (Cabo et al., 2006) and failing hearts (Poelzing and Rosenbaum, 2004; Akar et al., 2007). In an earlier computational study, we showed that, under conditions of uniform reduced excitability, heterogeneities in gap junction conductance could result in unidirectional block of non-premature impulses (Toure and Cabo, 2012). However, in cardiac patients, the areas of unidirectional block that lead to arrhythmias likely result from the interaction of spontaneously generated premature impulses and a substrate created by the remodeling of cell membrane properties and/or tissue structure (Baba et al., 2005; Cabo et al., 2006). In particular, the mechanisms of conduction and block of premature impulses in regions of heterogeneous gap junction conductance have not been fully characterized.

Our objective is to characterize the dynamics of propagation of premature impulses in the healing infarcted heart, and how those dynamics may result in unidirectional block in structurally remodeled myocardium with heterogeneities in gap junction conductance. To simulate the remodeling in cell membrane properties occurring after myocardial infarction, we use a previously developed ionic model of the action potential of cells from the canine epicardial border zone (Cabo and Boyden, 2003). To study the dynamics of propagation of the action potential, we use a sub-cellular computer model (Spach and Heidlage, 1995), which provides a realistic representation of the tissue microstructure including the natural variability in cell morphology and electrical connections through gap junctions. Sub-cellular models have been used to investigate the effect of cell size, gap junction remodeling, and myofibroblast proliferation on cardiac wave propagation (Hubbard et al., 2007; Cabo and Boyden, 2009; Toure and Cabo, 2010, 2012; Baum et al., 2012).

## Methods

### Numerical methods

We performed all simulations in a 2D monodomain model with governing equation: ∇ · ((1/(*S_v_R_i_C_m_*)) ∇ *V_m_*) = (*I_ion_/C_m_*) + ∂*V_m_*/∂*t*, where *V_m_* is the transmembrane potential (in mV), *I_ion_* is the ionic current (μ*A/cm*^2^), *R_i_* is the resistivity of the intracellular space, *S_v_* is the surface to volume ratio (2000 cm^−1^), and *C_m_* is the specific capacitance (1 μ*F/cm*^2^). Neumann (non-flow) boundary conditions were used. Membrane dynamics (*I_ion_*) were formulated by an ionic model of the action potential of canine epicardial infarcted border zone cell (IZ cell dynamics) (Cabo and Boyden, 2003). The density and kinetics of several ionic currents of IZ cells are markedly different from cells from non-infarcted canine epicardium (Cabo and Boyden, 2003). Earlier results indicate that I_Na_ density is a major determinant of propagation (or block) at sites of microstructural discontinuities (Toure and Cabo, 2012). When compared to cells from normal epicardium, IZ cells have a reduced I_Na_ density that results in a slower conduction velocity and a delayed recovery from inactivation of the Na channel that results in post-repolarization refractoriness.

**Figure 1:**
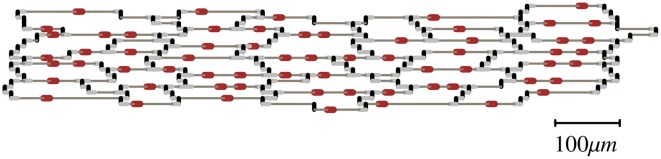
**Basic unit of the tissue architecture showing natural variability in cell size and shape as well as the location of gap junctions (Spach and Heidlage, 1995)**. Different types of gap junctions are shown on the cell membrane: plicate (black), interplicate (gray), and combined plicate (red).

We used the tissue architecture in Figure 1 as the basis to create other architectures with different cell-to-cell gap junctional conductance (*Gj*) (Spach and Heidlage, 1995). The basic unit in Figure 1 was replicated to create preparations of any size. That tissue architecture is a realistic representation of the natural variability in cell size and shape as well as the location of gap junctions. Each myocyte was electrically connected to neighboring myocytes only at the gap junctions. Three different types of gap junctions were simulated in the model: plicate (0.5 μS), interplicate (0.33 μS) and combined plicate gap junctions (0.062 μS) (Figure 1) (Spach and Heidlage, 1995). Plicate gap junctions, which are located in the plicate region of the intercalated disks, were simulated by resistors connecting cells electrically in the direction of the fiber orientation (longitudinally). Interplicate gap junctions, which are located in the interplicate region of the intercalated disks, were simulated by resistors connecting cells electrically in the direction transverse to the fiber orientation. Combined plicate gap junctions, which represent small intercalated disks located on the lateral membrane, between the larger intercalated disks containing the plicate and interplicate gap junctions, were simulated by resistors connecting cells electrically in the direction transverse to the fiber orientation. To study the effect of *Gj* heterogeneities on propagation, we used the basic tissue architecture, with different regions having different cell-to-cell *Gj* while the location of gap junctions was preserved. The regions with different cell-to-cell *Gj* were created by modifying the original conductance of the three types of gap junctions in the same proportion. The resistivity of the cytoplasm was 150 Ω *cm*. Cells were discretized with a space step of 10 μ*m* in both longitudinal and transverse directions. Each discretized cell element consisted of two square membrane surfaces, each one having a surface area of 100 μ*m*^2^, separated by a depth of 10 μ*m*, with a volume of 1000 μ*m*^3^. Those values result in a surface to volume ratio (*S_v_*) of 0.2 μ*m*^−1^ (=2000 *cm*^−1^). In all simulations *S_v_* was kept constant. The governing equation was integrated using the semi-implicit Crank-Nicholson method with a time step of 5 μs.

### Simulation protocols

We studied the dynamics of propagation of premature impulses in uniform preparations having the same average cell-to-cell *Gj* and in preparations with a structural discontinuity having different average cell-to-cell *Gj* in the region proximal to the discontinuity and in the region distal to the discontinuity. Flat wave fronts were initiated at the boundary of the proximal region of the preparation by an externally applied stimulus current (2x diastolic threshold). Propagation in all simulations was longitudinal, i.e., in the direction of the fiber orientation. The basic stimulation train consisted of 10 stimuli (S1) with a basic cycle length (BCL or S1S1) of 250 ms, after which single (S2), and double premature (S3) impulses with different coupling intervals were applied. The size of the preparations was 10 mm × 2.5 mm, and 7.5 mm × 2.5 mm, obtained by replication of the basic unit in Figure 1 to the appropriate size.

We also calculated λ for tissue architectures with different cell-to-cell *Gj*. λ was calculated from the spatial decay of *V_m_*, five membrane time constants (40 ms) after one end of the preparation was clamped at −65 *mV*.

## Results

### Propagation of single premature impulses (S2) in the infarcted heart

**Figure 2:**
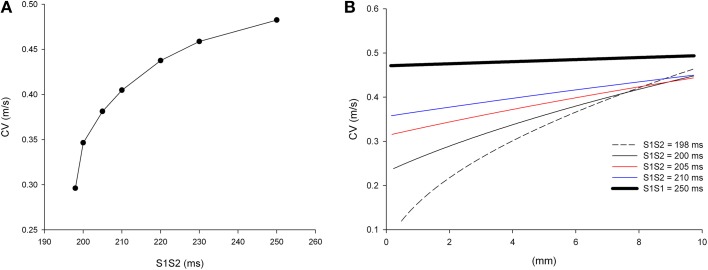
**Longitudinal conduction velocity (CV) of single premature impulses with different coupling intervals (S1S2) in a uniform infarcted myocardium preparation with average cell-to-cell coupling *Gj* = 0.41 μS. (A)** Average longitudinal CV in the preparation for different coupling intervals. **(B)** Local longitudinal CV as the impulse propagates away from the stimulation site (*x* = 0 mm). Size of the preparation: 10 × 2.5 mm.

It is well-known that the conduction velocity (CV) of premature impulses decreases with the coupling interval between the premature impulse and the last impulse of the basic train (S1S2). Figure 2A shows the CV restitution curve for single premature impulses after a basic train with a S1S1 coupling interval of 250 ms in a computer model of the infarcted heart with average cell-to-cell coupling *Gj* = 0.41 μS. The average CV of the most premature impulse that propagates (S1S2 = 198 ms) is 0.30 m/s, while the average CV of the basic impulse (S1S1 = 250 ms) is 0.48 m/s.

The CV of single premature impulses is not constant but it increases as the premature impulse propagates away from the stimulation site. Figure 2B shows the local CVs of premature impulses with S1S2 coupling intervals ranging from 198 ms (the shortest coupling interval that elicits a propagated response) to 250 ms. For S1S2 = 198 ms, CV varies from 0.12 m/s close to the stimulation site (*x* = 0 mm in Figure 2B) to 0.45 m/s 10 mm away from the stimulation site (*x* = 10 mm in Figure 2B). As the coupling interval of the premature impulses increases the changes in CV during propagation decrease. For example for the basic impulse in the train (S1S1 = 250 ms; thick black line in Figure 2B), CV close to the stimulation site is 0.47 m/s, and CV 10 mm away from the stimulation site is 0.49 m/s.

**Figure 3:**
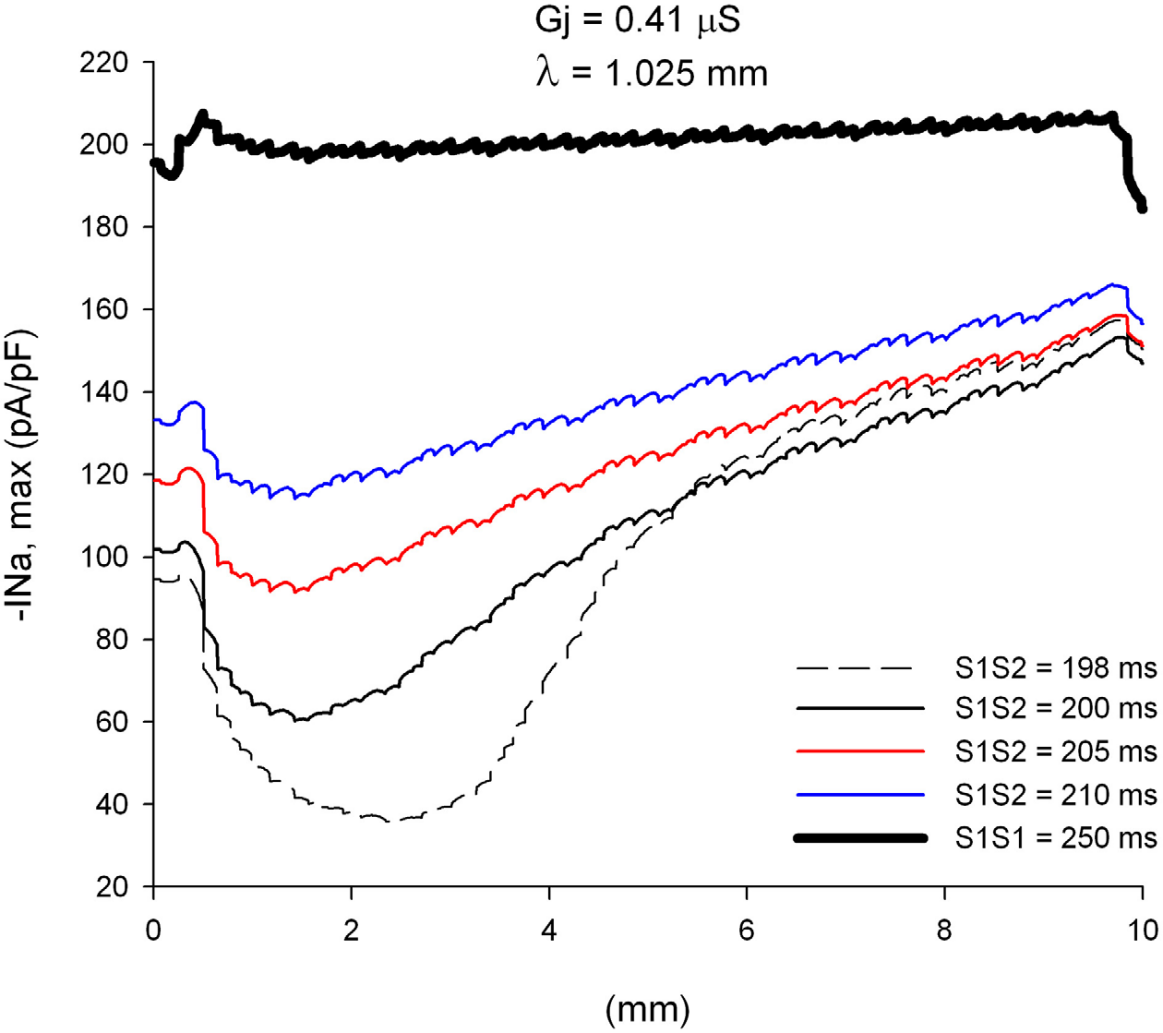
**Changes in I_Na_ current peak for single premature impulses, with different coupling intervals (S1S2), as the impulses propagate away from the stimulation site (*x* = 0 mm), in a uniform preparation with average cell-to-cell coupling *Gj* = 0.41 μS**. The dashed plot (S1S2 = 198 ms) is the shortest coupling that resulted in a propagated response. Size of the preparation: 10 × 2.5 mm.

The changes in CV of premature impulses are caused by the dynamics of recovery from inactivation of the sodium channel. Figure 3 shows how the absolute value of the peak I_Na_ current changes (|I_Na,max_|), along a line in the center of the preparation, as the premature impulse propagates away from the stimulation site (*x* = 0 mm in Figure 3) for different coupling intervals. For S1S2 = 198 ms, |I_Na,max_| first decreases to reach a minimum of about 36 pA/pF about 2.5 mm (~2.5 λ) away from the stimulation site and then increases steadily as the premature impulse propagates. This minimum identifies the location where the premature impulse is more vulnerable to block. The fact that the minimum |I_Na,max_| occurs about 2.5 λ from the stimulation site will have some implications for the conditions for block at sites of structural remodeling (see below). The steady increase in |I_Na,max_| (after having reached a minimum) correlates well with the increase in local CV as propagation proceeds shown in Figure 2B. Note in Figure 3 that the boundaries of the preparation have an effect on the value of |I_Na,max_|: the initiation of a propagating impulse by an external electrical stimulus affects the values of |I_Na,max_| within ~0.5 mm of the site of initiation (*x* = 0 mm); the rapid decrease of |I_Na,max_| at the end of the preparation (*x* = 10 mm) is a consequence of the rapid increase in V_m_ caused by the collision of the propagating wave with the sealed boundary preventing I_Na_ from full activation (Spach and Kootsey, 1985).

**Figure 4:**
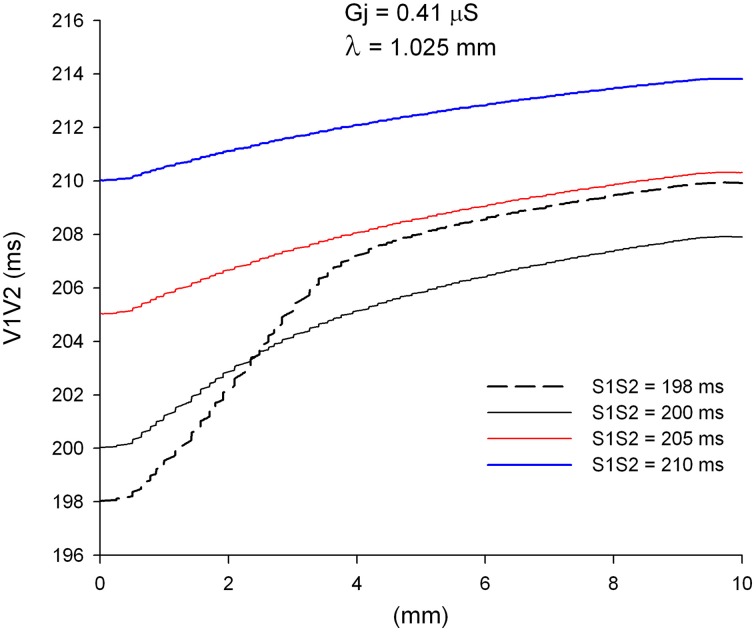
**Change in coupling interval between single premature impulses and the last impulse of the basic train (V1V2), for different coupling intervals (S1S2), as premature impulses propagate away from the stimulation site (*x* = 0 mm)**. The dashed plot (S1S2 = 198 ms) is the shortest coupling that resulted in a propagated response. Uniform preparation with average cell-to-cell coupling *Gj* = 0.41 μS. Size of the preparation: 10 × 2.5 mm.

An important consequence of the dynamics of propagation of single premature impulses shown in Figures 2, 3 is that as single premature impulses propagate away from the site of initiation, they become less and less premature. Figure 4 shows the coupling interval of premature impulses with respect to the last impulse of the basic train (V1V2) as propagation proceeds away from the stimulation site (*x* = 0 mm). For example, a premature impulse initiated with a coupling interval of S1S2 = 200 ms at the simulation site, will have a V1V2 coupling interval of 206 ms at a distance of 5 mm from the stimulation site, and 208 ms at a distance of 10 mm from the stimulation site. This is a consequence of the difference in CV between the premature and the last basic impulse (Figure 2). Note also that premature impulses initiated with a very short coupling interval (S1S2 = 198 ms in Figure 4) can result in a longer V1V2 coupling interval far away from the stimulation site (compare V1V2 for S1S2 = 198 ms and S1S2 = 200 ms).

### Effect of structural remodeling on propagation of single premature impulses (S2)

**Figure 5:**
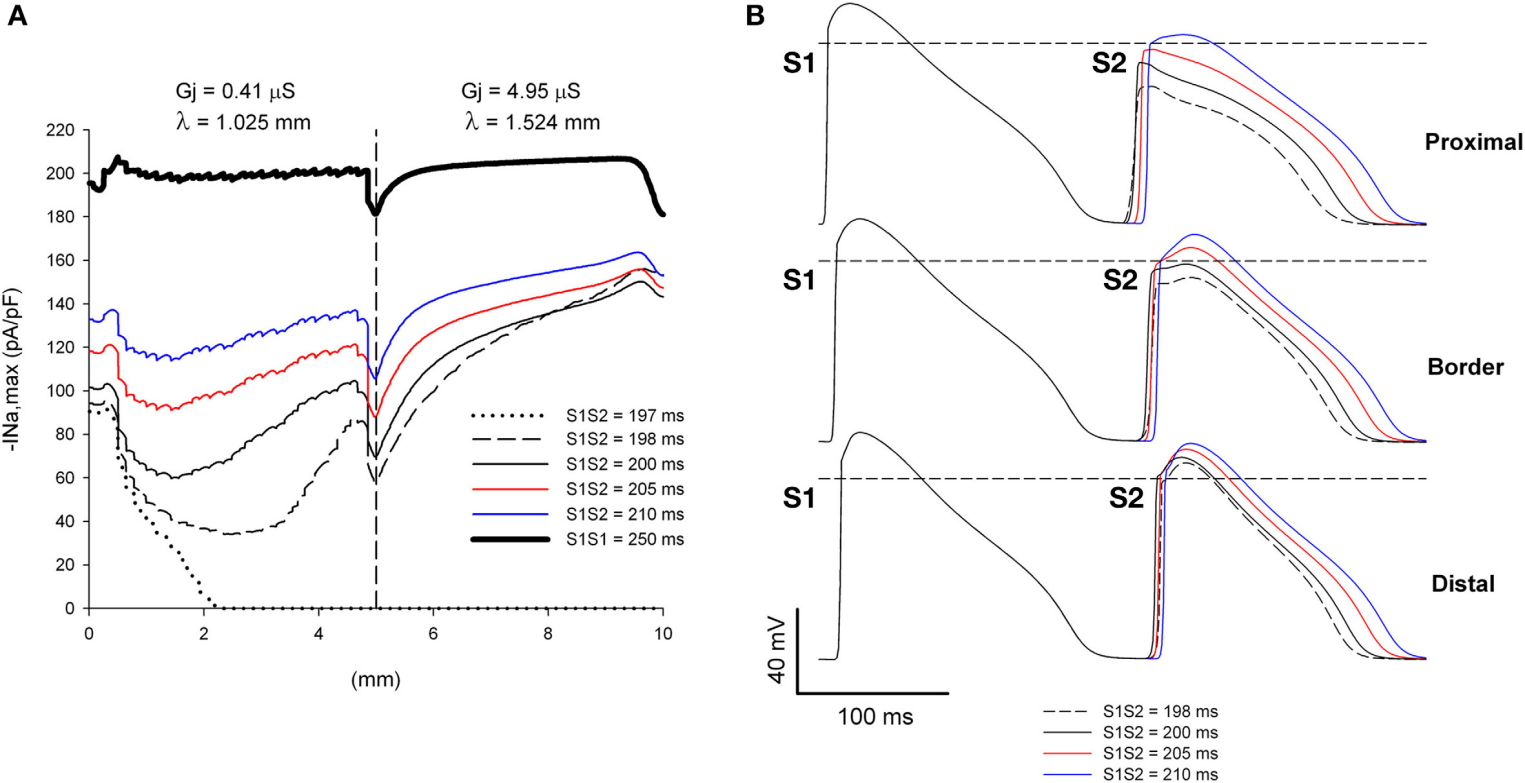
**Single premature impulses propagate through a structural heterogeneity when initiated far (5 mm away) from the heterogeneity. (A)** Changes in I_Na_ current peak for single premature impulses, with different coupling intervals (S1S2), as the impulses propagate away from the stimulation site (*x* = 0 mm), in a preparation with a structural heterogeneity with average cell-to-cell coupling *Gj* = 0.41 μS in the proximal side and *Gj* = 4.95 μS in the distal side. The dashed vertical line indicates the location of the heterogeneity. **(B)** Action potentials calculated in the proximal side (2.5 mm from the border), at the border and in the distal side (2.5 mm from the border). Horizontal dashed line indicates a transmembrane potential of 0 mV. The dashed plot (S1S2 = 198 ms) is the shortest coupling that resulted in a propagated response. Size of the preparation: 10 × 2.5 mm (proximal side: 5 mm; distal side: 5 mm). Note that all single premature impulses propagate through the structural heterogeneity.

We have reported earlier that, in normal myocardium, under conditions of reduced excitability (70% reduction in maximum sodium channel conductance), propagation of action potentials blocks at sites where the space constant in the direction of propagation increases by >40% (Toure and Cabo, 2012). Therefore, we analyzed the dynamics of propagation of premature impulses through heterogeneities in gap junctional conductance which result in increases of space constant of more than 40% in the direction of propagation. Figure 5 shows |I_Na,max_| during propagation of premature impulses through a heterogeneity in gap junctional conductance (λ_distal_/λ_proximal_ = 1.49 or an increase of 49% in the direction of propagation) which is identified in Figure 5A by the vertical dashed line. The refractory period is 197 ms (a premature with S1S2 = 197 ms fails to propagate in the proximal side of the heterogeneity). Note in Figures 5A,B that all premature impulses (S2) that propagate in the proximal side (S1S2 ≥ 198 ms), also propagate through the heterogeneity (propagation initiated at *x* = 0 mm). The effect of the heterogeneity is to reduce |I_Na,max_| by ~30 pA/pF for the more premature impulses and by ~20 pA/pF for the basic impulse of the stimulating train (S1S1 = 250 ms). At the interface, as the downstream impedance decreases in tissue with higher cell-to-cell coupling, the stimulatory efficacy of the wave front head decreases due to current dissipation, a phenomenon known as source/sink mismatch. The drop in the absolute value of peak I_Na_ caused by the heterogeneity is not sufficient to bring |I_Na,max_| below the value that is necessary to block propagation, and that is why all premature impulses propagate through the heterogeneity. For example, for S1S2 = 198 ms, after the premature impulses reaches a minimum |I_Na,max_| of 34 pA/pF 2.5 mm away from the stimulation site, |I_Na,max_| increases to ~ 86 pA/pF as the premature impulse propagates, which is reduced by the source/sink mismatch at the heterogeneity to ~ 58 pA/pF, a value which is still sufficient to sustain propagation.

**Figure 6:**
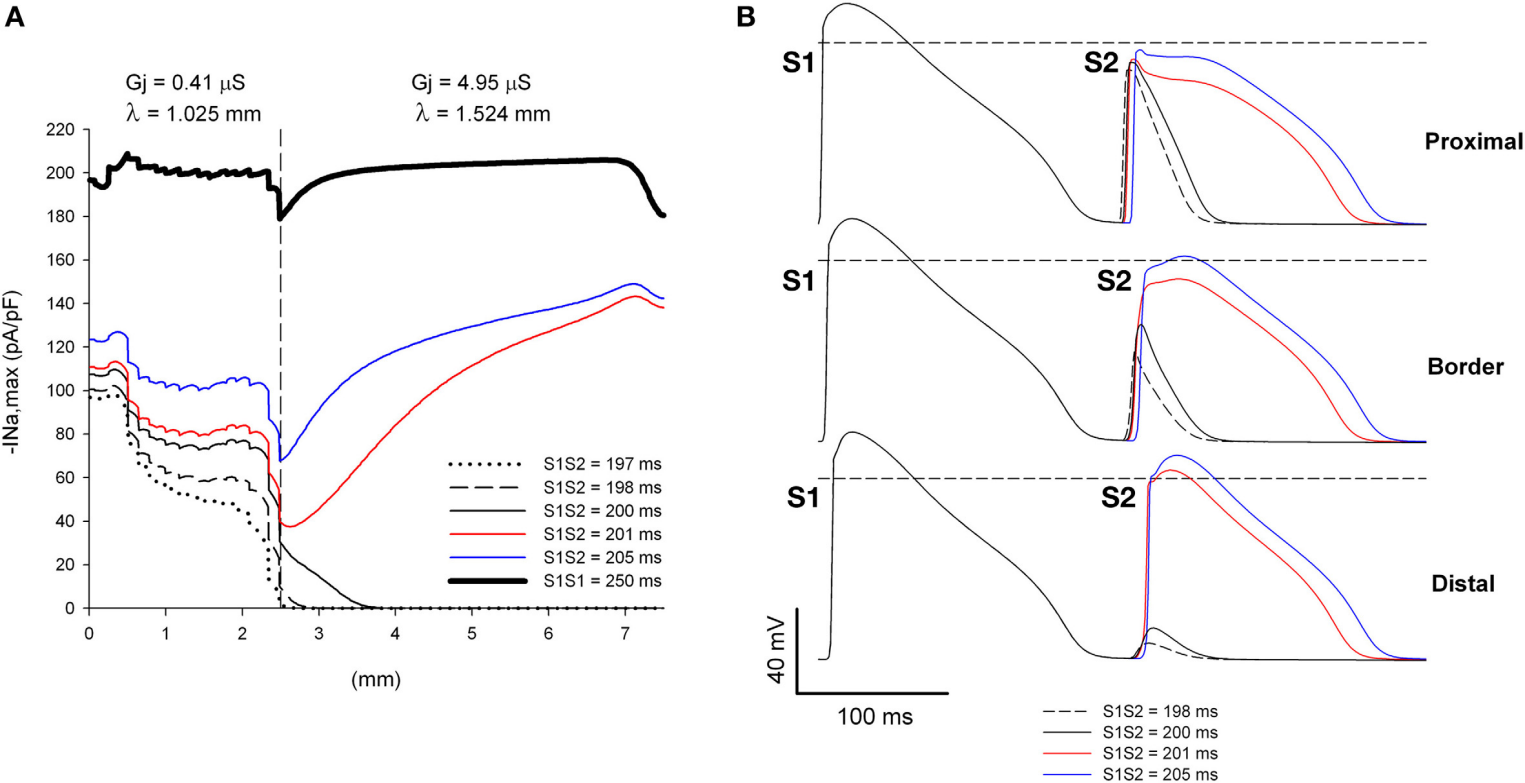
**Single premature impulses may block at a structural heterogeneity when initiated close (2.5 mm away) to the heterogeneity. (A)** Changes in I_Na_ current peak for single premature impulses, with different coupling intervals (S1S2), as the impulses propagate away from the stimulation site (*x* = 0 mm), in a preparation with a structural heterogeneity with average cell-to-cell coupling *Gj* = 0.41 μS in the proximal side and *Gj* = 4.95 μS in the distal side. The dashed vertical line indicates the location of the heterogeneity. **(B)** Action potentials calculated in the proximal side (1.5 mm from the border), at the border and in the distal side (2.5 mm from the border). Horizontal dashed line indicates a transmembrane potential of 0 mV. The dashed plot (S1S2 = 198 ms) is the shortest coupling that resulted in a propagated response (in the proximal side). Size of the preparation: 7.5 × 2.5 mm (proximal side: 2.5 mm; distal side: 5 mm). Note that single premature impulses with S1S2 = 198–200 ms block at the structural heterogeneity.

If the initiation of premature impulses occurs closer to the heterogeneity, premature impulses block for a range of coupling intervals (Figure 6). In Figure 6 initiation of premature impulses occurs 2.5 mm (~ 2.5 λ) away from the same heterogeneity in gap junction conductance shown in Figure 5. Premature impulses initiated with coupling intervals S1S2 of 198–200 ms block at the heterogeneity (the window of vulnerability to block is 2–3 ms) (Figures 6A,B). Note that premature impulses at those same coupling intervals do not block when the premature impulses are initiated 5 mm (~ 5 λ) away from the heterogeneity (Figure 5). The mechanism of block relates to the dynamics of propagation of premature impulses shown in Figure 3. Premature impulses show a minimum |I_Na,max_|, and are more susceptible to block, at a distance ~ 2 mm from the stimulation site (Figures 3, 5); as propagation proceeds, |I_Na,max_| increases. The heterogeneity in gap junction conductance that results in a sudden increase in space constant in the direction of propagation causes a drop in |I_Na,max_| (Figure 5A). If that drop occurs when the premature impulse is more vulnerable, the value of |I_Na,max_| may decrease below the value that is necessary to sustain propagation, and the premature impulse may block (S1S2 = 198–200 ms in Figure 6A). The critical value of |I_Na,max_| for propagation is ~ 40 pA/pF (the minimum for S1S2 = 201 ms in Figure 6A). If the drop occurs when |I_Na,max_| has partially recovered from its minimum, the drop is not sufficient to bring |I_Na,max_| below the critical value for propagation, and the premature impulse propagates through the heterogeneity (S1S2 = 198 ms and 200 ms in Figure 5A).

**Figure 7:**
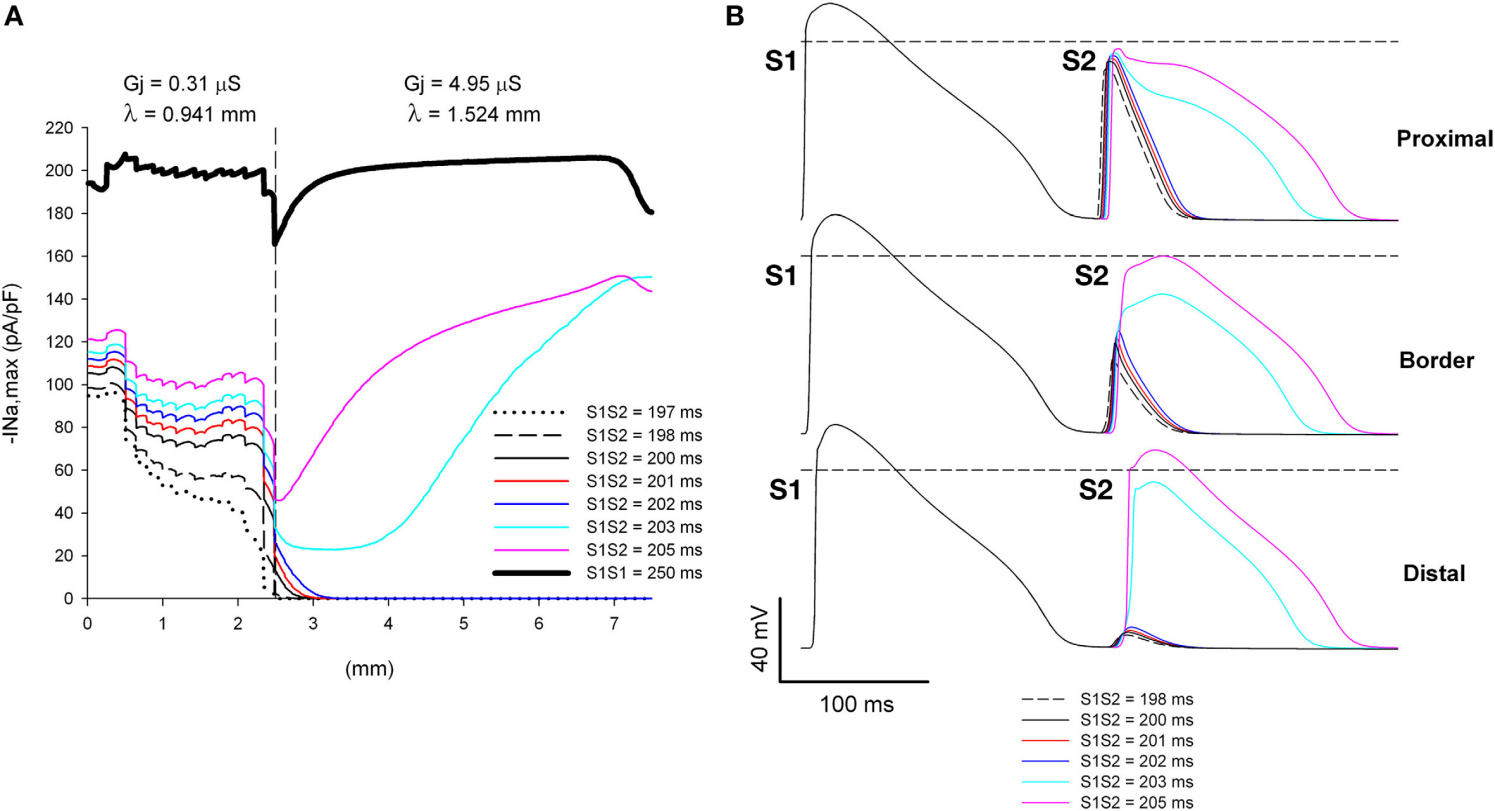
**Increasing the discontinuity in *Gj* at the heterogeneity increases the range of coupling intervals for which single premature impulses block at a structural heterogeneity when initiated close (2.5 mm away) to the heterogeneity. (A)** Changes in I_Na_ current peak for single premature impulses, with different coupling intervals (S1S2), as the impulses propagate away from the stimulation site (*x* = 0 mm), in a preparation with a structural heterogeneity with average cell-to-cell coupling *Gj* = 0.31 μS in the proximal side and *Gj* = 4.95 μS in the distal side. The dashed vertical line indicates the location of the heterogeneity. **(B)** Action potentials calculated in the proximal side (1.5 mm from the border), at the border and in the distal side (2.5 mm from the border). Horizontal dashed line indicates a transmembrane potential of 0 mV. The dashed plot (S1S2 = 198 ms) is the shortest coupling that resulted in a propagated response (in the proximal side). Size of the preparation: 7.5 × 2.5 mm (proximal side: 2.5 mm; distal side: 5 mm). Note that single premature impulses with S1S2 = 198–202 ms block at the structural heterogeneity.

The window of vulnerability to block increases when the difference in *Gj* between the distal and proximal sides of the heterogeneity (and consequently the ratio of the space constants) increases. Figure 7 shows the dynamics of propagation of premature impulses through a heterogeneity with λ_distal_/λ_proximal_ = 1.62. The increase in the source/sink mismatch results in a larger drop in |I_Na,max_| at the heterogeneity (compare Figure 7A with Figure 6A). Premature impulses with coupling intervals S1S2 = 198–202 ms block at the heterogeneity increasing the window of vulnerability to block to 4–5 ms (which is larger than in Figure 6). Note the latency in the recovery of |I_Na,max_| for S1S2 = 203 ms, which indicates that the premature impulse hovers around the threshold of |I_Na,max_| necessary for propagation in the distal side (~ 24 pA/pF). For the *Gj* heterogeneity in Figure 7, when initiation of premature impulses occurred 5 mm away from the heterogeneity, similarly to what occurred in Figure 5, all premature impulses propagated through the heterogeneity (not shown).

### Propagation of double premature impulses (S3) in the infarcted heart

**Figure 8:**
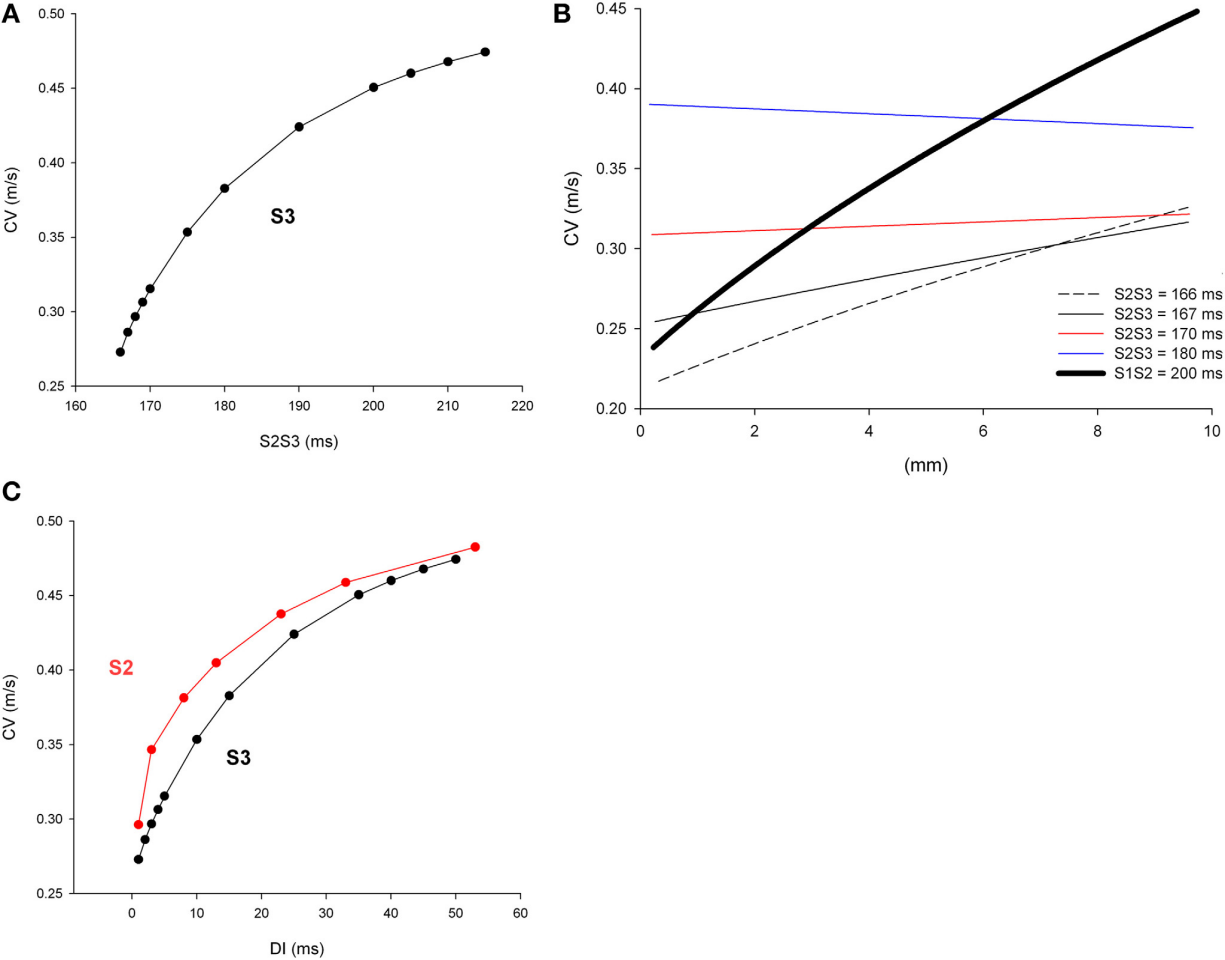
**Longitudinal conduction velocity (CV) of double premature impulses with different coupling intervals (S2S3), after a single premature with a coupling interval S1S2 = 200 ms, in a uniform infarcted myocardium preparation with average cell-to-cell coupling *Gj* = 0.41 μS**. **(A)** Average longitudinal CV in the preparation for different coupling intervals. **(B)** Local longitudinal CV as the impulse propagates away from the stimulation site (*x* = 0 mm). **(C)** Comparison of longitudinal CV restitution curves for single (S2) and double (S3) premature impulses. The diastolic interval (DI) is defined as (S1S2—Refractory period of S1) for S2 and (S2S3—Refractory period of S2) for S3 impulses. Size of the preparation: 10 × 2.5 mm.

Figure 8A shows the CV of double premature impulses (S3) initiated after a first premature (S2) with a coupling interval S1S2 = 200 ms with different S2S3 coupling intervals. As expected, and similarly to what occurred for single premature impulses (Figure 2A), average CV decreases with S2S3 coupling interval. Figure 8B shows the local CV as the S3 impulse propagate away from the stimulation site. CVs change less as the S3 premature propagates (Figure 8B) than when S2 impulses propagate (Figure 2B). Compare for example the changes in CV occurring when the S2 impulse with coupling interval S1S2 = 200 ms propagates (thick black line in Figure 8B) with the changes occurring when a S3 impulse with a coupling interval S2S3 = 166 ms propagates (black dashed line in Figure 8B). In contrast to what occurred during propagation of single premature impulses that show a monotonic increase in CV with propagation, during propagation of double premature impulses (S3) CV can decrease as the wave front propagates (coupling interval S2S3 = 180 ms in Figure 8B). For comparison purposes, Figure 8C shows both the CV restitution curves for single (S2) and double (S3) premature impulses. The maximum slope of the CV restitution curve for S3 is smaller than for S2, and for all diastolic intervals (DI), the CV for S3 premature impulses is smaller than for S2 premature impulses. However, the CV of a S3 impulse with a big DI is larger than the CV of a S2 impulse with a small DI (see below). For example the CV of S3 with *DI* = 25 ms is 0.42 m/s while the CV of S2 with *DI* = 3 ms is 0.35 m/s.

**Figure 9:**
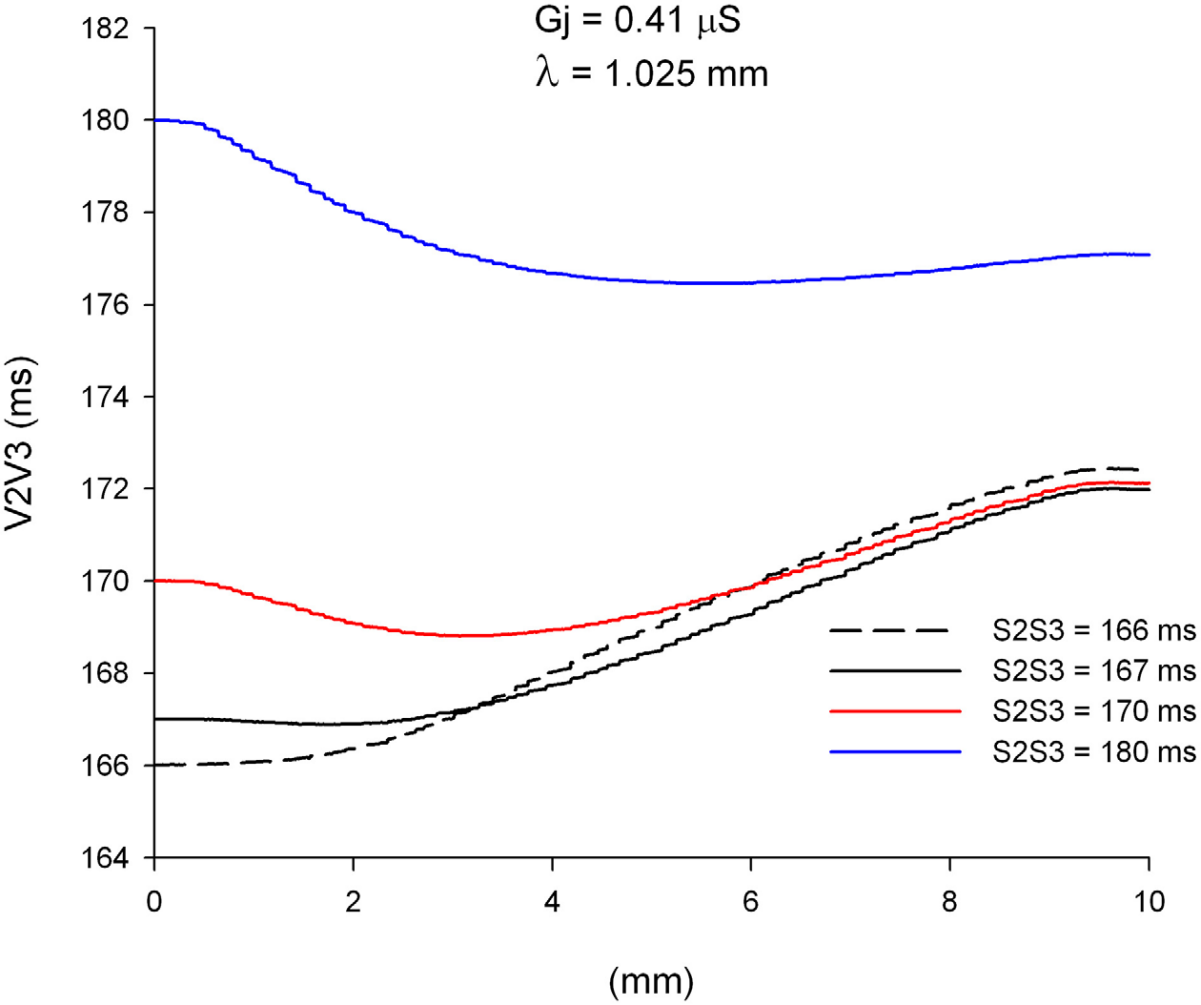
**Change in coupling interval between double premature impulses and a single premature with S1S2 = 200 ms (V2V3), for different coupling intervals (S2S3), as double premature impulses propagate away from the stimulation site (*x* = 0 mm)**. Uniform preparation with average cell-to-cell coupling *Gj* = 0.41 μS. Size of the preparation: 10 × 2.5 mm.

We have shown earlier that single premature impulses become less premature as the impulse propagates away from the stimulation site (Figure 4) because the CV of single premature impulses (S2) is slower than the CV of the last basic impulse of the train (S1) (see CV restitution curve in Figure 2A). However, this is not necessarily the case for propagation of double premature impulses. Figure 9 shows the coupling interval of double premature impulses (V2V3) as the impulse propagates away from the stimulation site. For S2S3 = 180 ms, the coupling interval of the double premature impulse (V2V3) decreases as the distance from the stimulation site increases (in contrast to what happened for single premature impulses in Figure 4). This is a consequence of the fact that the CV of the double premature impulse (S2S3 = 180 ms) is faster than the CV of the single premature impulse (S1S2 = 200 ms) (Figures 8A,B). The average CV for the single premature (S1S2 = 200 ms; *DI* =3 ms in S2 plot in Figure 8B) is ~0.35 m/s, and the average CV for the double premature (S2S3 = 180 ms; *DI* = 15 ms in S3 plot in Figure 8B) is ~0.38 m/s. Note also the differences in CV for single and double premature impulses in Figure 8B. Only for double premature impulses with a small S2S3 coupling interval (and small DI) the premature impulse becomes less premature as the distance from the stimulation site increases. For example, for S2S3 = 166 ms (black dashed line in Figure 9), the coupling interval of the double premature impulse 10 mm away from the stimulation site is V2V3 = 172 ms; this is a consequence of the fact that the CV of the double premature impulse is less than the CV of the single premature (Figure 8B).

**Figure 10:**
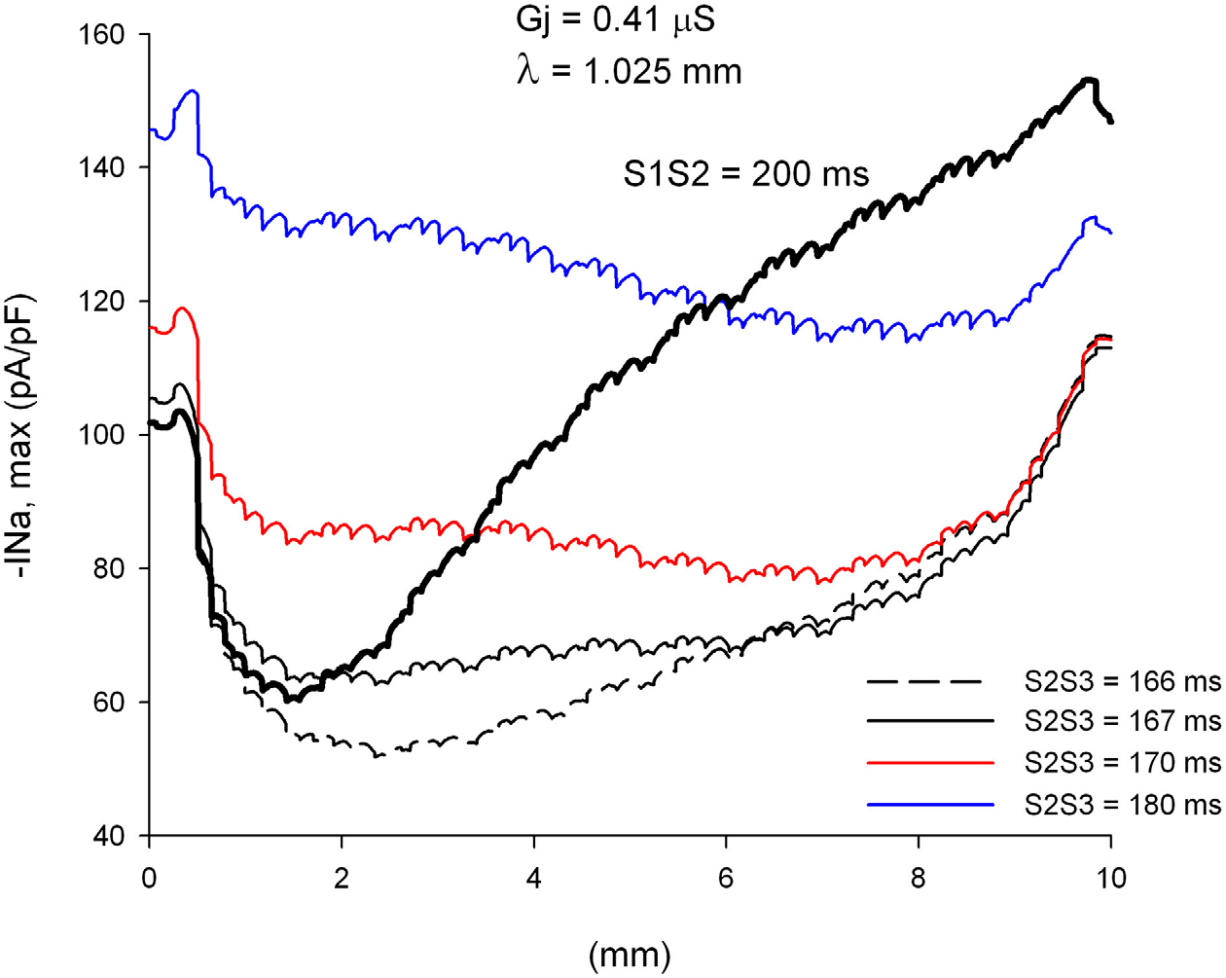
**Changes in I_Na_ current peak for double premature impulses with different coupling intervals (S2S3), initiated after a single premature with S1S2 = 200 ms, as the impulses propagate away from the stimulation site (*x* = 0 mm), in a uniform preparation with average cell-to-cell coupling *Gj* = 0.41 μS**. Also shown for comparison are the changes in I_Na_ current peak for the single premature impulse (S1S2 = 200 ms; thick black line; also shown in Figure 3). Size of the preparation: 10 × 2.5 mm.

We have shown earlier that single premature impulses reach a minimum |I_Na,max_| about 2 mm (2λ) from the stimulation site and then recover as propagation proceeds (Figure 3). For double premature impulses, |I_Na,max_| does not recover as fast as for single premature impulses and low values of |I_Na,max_| persist further away from the stimulation site (Figure 10). For double premature impulses, there is not a marked minimum from which |I_Na,max_| recovers quickly. For S2S3 = 166 ms (*DI* = 1 ms), low values of |I_Na,max_| persist 6 mm away from the stimulation site, and its recovery is very slow. Compare with the dynamics of recovery of S1S2 = 200 ms (*DI* = 3 ms), which is also shown for reference in Figure 10 (thick black line). For S2S3 = 167, 170, and 180 ms there is no evidence of recovery of |I_Na,max_| with distance from the stimulation site except when the impulses reach the boundary of the preparation (*x* = 10 mm). In fact, for S2S3 = 170 and 180 ms, |I_Na,max_| decreases as the impulse propagates away of the simulation site.

### Effect of structural remodeling on propagation of double premature impulses (S3)

**Figure 11:**
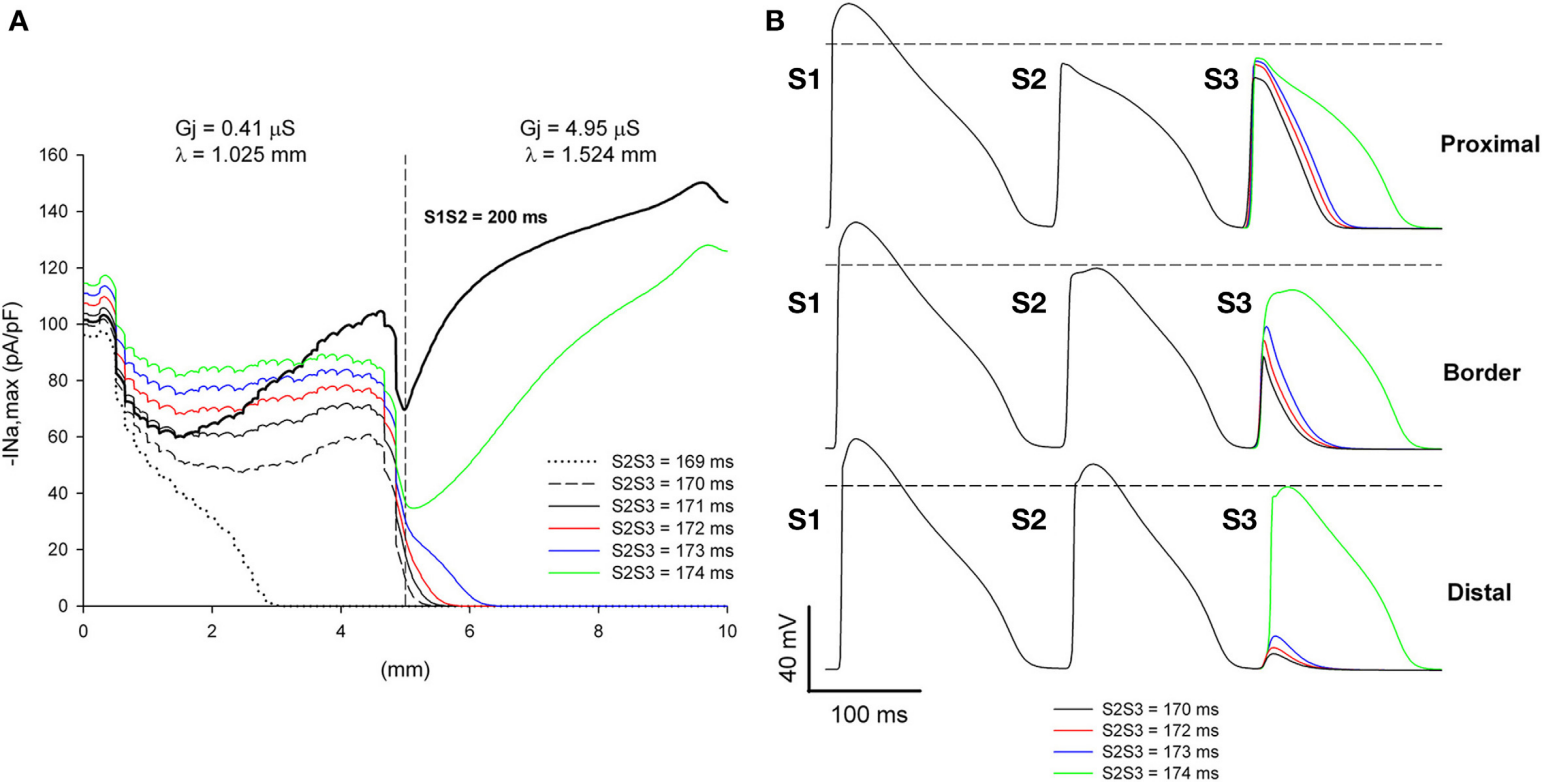
**Double premature impulses block at a structural heterogeneity even when initiated far (5 mm away) from the heterogeneity. (A)** Changes in I_Na_ current peak for double premature impulses, with different coupling intervals (S2S3), as the impulses propagate away from the stimulation site (*x* = 0 mm), in a preparation with a structural heterogeneity with average cell-to-cell coupling *Gj* = 0.41 μS in the proximal side and *Gj* = 4.95 μS in the distal side. Also shown, for reference, are the changes in I_Na_ current peak for the single premature impulse (S1S2 = 200 ms) initiated before the double premature impulses. The dashed vertical line indicates the location of the heterogeneity. **(B)** Action potentials calculated in the proximal side (2.5 mm from the border), at the border and in the distal side (2.5 mm from the border). Horizontal dashed line indicates a transmembrane potential of 0 mV. The dashed plot (S2S3 = 170 ms) is the shortest coupling that resulted in a propagated response in the proximal side. Size of the preparation: 10 × 2.5 mm (proximal side: 5 mm; distal side: 5 mm). Note that double premature impulses with coupling intervals S2S3 = 170–173 ms block at the structural heterogeneity.

We have shown earlier that, for a heterogeneity with λ_distal_/λ_proximal_ = 1.49 (or an increase of 49% in the direction of propagation), single premature impulses with the shortest coupling interval propagate through the heterogeneity when they are initiated far away (5 mm) from the heterogeneity (Figure 5). This is a consequence of the recovery of |I_Na,max_| of single premature impulses as they propagate away from the site of initiation. However, propagating double premature impulses do not show such a recovery in |I_Na,max_|, and low values of |I_Na,max_| persist far way form the stimulation site (Figure 10). As a result, and in contrast to what occur with single premature impulses, double premature impulses initiated 5 mm away from a heterogeneity with λ_distal_/λ_proximal_ = 1.49 block for a range of S2S3 coupling intervals from 170 to 173 ms (window of vulnerability is 4–5 ms) (Figure 11). The mechanism of block is the drop in |I_Na,max_| caused by the source/sink mismatch at the heterogeneity. For single premature impulses (thick black line in Figure 11), the drop occurs after |I_Na,max_| had recovered and the drop is not sufficient to reduce it below a value that cannot sustain propagation. In contrast, for double premature impulses (with short coupling intervals), |I_Na,max_| does not recover sufficiently with propagation and the drop in |I_Na,max_| at the heterogeneity decreases it to a value that cannot sustain propagation.

**Figure 12:**
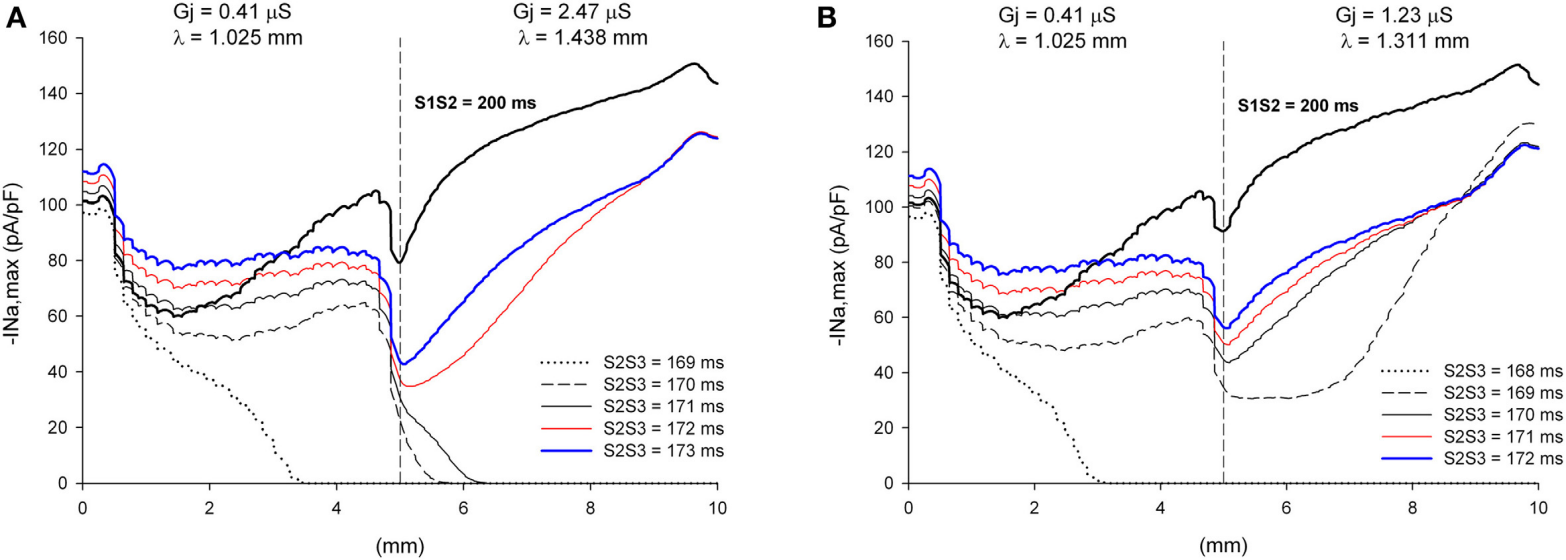
**Block at a structural heterogeneity depends on the difference in *Gj* between the proximal and the distal sides of the heterogeneity**. Changes in I_Na_ current peak for double premature impulses, with different coupling intervals (S2S3), as the impulses propagate away from the stimulation site (*x* = 0 mm), in a preparation with a structural heterogeneity with average cell-to-cell coupling *Gj* = 0.41 μS in the proximal side and *Gj* = 2.47 μS in the distal side (λ_distal_/λ_proximal_ = 1.40, **A**) and *Gj* = 0.41 μS in the proximal side and *Gj* = 1.23 μS in the distal side (λ_distal_/λ_proximal_ = 1.3, **B**). Also shown, for reference, are the changes in I_Na_ current peak for the single premature impulse (S1S2 = 200 ms) initiated before the double premature impulses. The dashed vertical line indicates the location of the heterogeneity. Note that when λ_distal_/λ_proximal_ = 1.4 the window of vulnerability is 2–3 ms (S2S3 = 170–171 ms, **A**) and that when λ_distal_/λ_proximal_ = 1.3 there is no block at the heterogeneity for any S2S3 coupling interval **(B)**.

The window of vulnerability to block decreases when the difference in cell-to-cell *Gj* between the proximal and the distal side decreases. For λ_distal_/λ_proximal_ = 1.40, the window of vulnerability decreases to 2–3 ms (Figure 12A), and for λ_distal_/λ_proximal_ = 1.3 there is no block at the heterogeneity for any coupling interval (Figure 12B). As the difference in *Gj* between the proximal and distal side decreases, so does the drop in |I_Na,max_| caused by the *Gj* heterogeneity (Figures 11, 12).

## Discussion

We have shown that |I_Na,max_|, prematurity (i.e., coupling interval with the previous impulse), and CV of premature impulses change dynamically as they propagate away from the site of initiation, and that there are fundamental differences between the dynamics of propagation of single (S2) and double premature impulses (S3). Single premature impulses become less premature, recover their excitability (|I_Na,max_| increases) and CV increase as propagation proceeds. As a consequence it is unlikely that single premature impulses will block at structural heterogeneities causing source/sink mismatch unless the site of origin of the impulse is close (within 2.5 λ) to the site of the heterogeneity or a transition between thin and thick fibers. In contrast, double premature impulses could become more premature, they do not recover their excitability (low values of |I_Na,max_| persist far away from the site of initiation) and their CV could decrease as propagation proceeds. Those dynamics make it more likely that double premature impulses block at sites of structural heterogeneities than single premature impulses. While experimental and clinical electrophysiologists have reported for many years that the use of multiple premature impulses during programmed electrical stimulation increases the chances of initiation of ventricular tachycardia (Wit and Janse, 1993), our study provides novel insights into the dynamics of propagation of premature impulses and a mechanistic explanation of the conditions for block at sites of microstructural heterogeneities. The dynamic changes in CV of premature impulses as propagation proceeds away from the site of initiation implies that measurement techniques that provide local CVs would be more suitable to quantify propagation of premature impulses than techniques that provide global CVs (Linnenbank et al., 2014).

### Initiation of arrhythmias by external premature stimulation

Regions of heterogeneous Cx43 expression and gap junction conductance have been described in infarcted and failing hearts (Poelzing and Rosenbaum, 2004; Cabo et al., 2006; Akar et al., 2007), can lead to source/sink mismatch and block of propagation (Kleber and Rudy, 2004), and are likely to provide the “substrate” that leads to initiation of cardiac arrhythmias in diseased hearts. Ventricular tachycardia (VT) can be initiated in clinical electrophysiology laboratories in ~90% of the patients who suffer spontaneous episodes of VT (Wit and Janse, 1993). Several clinical studies (Wit and Janse, 1993) have shown that premature stimulation with a single premature stimulus can induce VT in 20–30% of the patients; two premature stimuli can increase that number by 55%, and the use of three premature impulses can further increase the number by 20%. All in all clinical evidence shows that the use of multiple premature impulses facilitates the initiation of VT. The failure of single premature impulses to initiate arrhythmias is generally explained by their inability to reach the site of the origin of the arrhythmia (Wit and Janse, 1993). Our results explain those experimental and clinical observations by the different dynamics of propagation of S2 and S3 premature impulses. S2 premature impulses may not be able to reach the site of origin of the arrhythmia (possibly a region with heterogeneous cell-to-cell *Gj*) because they recover their excitability relatively close to the stimulation site and they may not be sufficiently premature (Figure 5). In contrast, S3 premature impulses do not recover their excitability as fast as S2 impulses, and may be able to reach the site of origin of the arrhythmia with low values of |I_Na,max_| (Figure 11).

The site(s) where premature stimuli are applied during an electrophysiological study may determine whether clinical VTs are initiated or not. It has been reported that one or two premature stimuli initiated on the right ventricle may not induce all clinical VTs (Robertson et al., 1981; Morady et al., 1984). The yield of clinical VTs increases (more clinical VTs induced) when the same protocol is applied to the left ventricle (Robertson et al., 1981; Morady et al., 1984). It is possible that premature impulses are initiated closer to an area with structural heart disease during left ventricular stimulation than during right ventricular stimulation. That would be consistent with the results above demonstrating that single premature impulses may block at structural heterogeneities when the site of origin of the impulse is close to the heterogeneity (Figures 6, 7) but not when the site of origin is far away (Figure 5).

### Spontaneous initiation of arrhythmias by premature impulses

The patterns of spontaneous initiation of sustained VT in post-myocardial infarction patients can be classified according to the morphology and coupling interval of the premature impulse(s) that precedes the tachycardia (Berger et al., 1988; Roelke et al., 1994). In the first pattern, the premature impulses have a morphology that is similar to that of the VT and a long coupling interval with the last sinus beat. Given the long coupling interval of premature impulses and their morphological similarity to the tachycardia, it has been speculated that those premature impulses could be sinus beats entering an infarcted region, conducting slowly in that region and exiting it before the next sinus beat arrives to excite the rest of the ventricle. In the second pattern, the premature impulses have a morphology that is different from that of the VT and a shorter coupling interval with the last sinus beat.

The success of single premature impulses in spontaneously initiating VT depends on the pattern of initiation. For the first pattern (similar morphology, long coupling), ~70% of VT are initiated by single premature impulses. For the second pattern (different morphology, shorter coupling), only ~16% of VT are initiated by single premature impulses and the rest by double or multiple premature impulses. While, for the first pattern, premature impulses are considered premature because they excite the ventricle prematurely (i.e., before the next sinus beat arrives), the long coupling intervals are much longer than the ventricular refractory period, and the dynamics of propagation of premature impulses described in this study most likely do not apply. However, for the second pattern, the shorter coupling intervals are likely closer to the refractory period, and the dynamics of propagation of premature impulses shown above explain why single premature impulses alone may not be successful in initiating VT, and two or more premature impulses could be necessary.

Microstructural heterogeneities caused by fibrosis can lead to abnormal propagation as a result of source/sink impedance mismatch in explanted hearts from patients with Brugada syndrome (Coronel et al., 2005; Hoogendijk et al., 2010). Even though the cellular mechanism of the heterogeneity in this study (cell-to-cell *Gj* remodeling) is not fibrosis, it is possible that the dynamics of propagation of premature impulses described here may play a role in the initiation of arrhythmias in Brugada syndrome patients.

### Cellular mechanisms of propagation and block at sites of microstructural heterogeneities causing source/sink impedance mismatch

In an earlier computational study, we found that, in a model of healthy canine epicardium under conditions of reduced excitability (uniform 70% reduction of maximum I_Na_ conductance), heterogeneities in gap junction conductance or cell size (both parameters of the cellular microstructure contribute to λ) cause unidirectional block when λ in the direction of propagation increases by at least 40% (Toure and Cabo, 2012). We have also shown that heterogeneities in myofibroblast density, whose paracrine effect results in heterogeneities in *Gj*, may also create a substrate leading to unidirectional block (Baum et al., 2012). In particular, block of premature impulses occurred at a heterogeneity between an area with a high (50%) density of myofibroblasts (λ ~ 1.02 mm) and an area without myofibroblasts (λ ~ 1.32 mm) (Baum et al., 2012). The results presented here are quantitatively consistent with those earlier results (Figures 11, 12), indicating that a sharp increase in λ by more than 30–40% in the direction of propagation may lead to block during premature stimulation or under conditions of reduced excitability. It seems that the threshold for block is independent of the cellular mechanism causing the change in space constant (gap junction conductance, cell size, myofibroblast density), and may apply to other situations in which cellular and/or tissue remodeling results in a change of λ For instance, it may explain the conditions for block at the border between two regions with different fiber alignments (Kudryashova et al., 2014). Block would be expected at a transition from a region where propagation occurs in the direction transverse to the fiber orientation (λ in the direction of propagation = λ_Trans_) to a region where propagation occurs in the direction longitudinal to the fiber orientation (λ in the direction of propagation = λ_Long_), if λ_Long_/λ_Trans_ > 1.40.

The density and kinetics of ionic currents are also important determinants of propagation of the action potential at sites of microstructural heterogeneities. Our results show that source/sink impedance mismatch at the site of the heterogeneity causes a decrease in I_Na_ (Figures 6, 7, 11, 12). When the value of I_Na_ at the site of the heterogeneity is close to the value necessary to sustain propagation, the L-type Ca current may determine whether propagation succeeds or not (Joyner et al., 1996; Rohr and Kucera, 1997; Rohr et al., 1997; Shaw and Rudy, 1997; Cabo et al., 2000). Consequently, we expect that modulation of the L-type Ca current will play a crucial role in determining successful propagation of premature impulses through microstructural heterogeneities caused by *Gj* remodeling in infarcted myocardium (Cabo et al., 2000).

### Restitution of the conduction velocity of premature impulses

The difference in the dynamics of propagation of single (S2) and double premature (S3) impulses is reflected in their CV restitution curves (Figure 8C). The pronounced maximum slope of the CV restitution curve for S2 indicates a faster recovery of excitability with diastolic interval and propagation. The smaller maximum slope of the restitution curve for S3 indicates a slower recovery of excitability with diastolic interval and propagation. The cumulative experimental and clinical evidence, as well as the computations presented here, indicate that double premature impulses are more pro-arrhythmic than single premature impulses. Therefore, we can argue that a decrease in the maximum slope in the CV restitution curve of a propagating impulse is indicative of an increased potential for block at structural heterogeneities. This is consistent with other reports demonstrating that less steep CV restitution curves were more likely to cause spiral break up (Qu et al., 1999), and that steep CV restitution curves prevent block in heterogeneous tissues (Sampson and Henriquez, 2001).

### Conflict of interest statement

The author declares that the research was conducted in the absence of any commercial or financial relationships that could be construed as a potential conflict of interest.
